# Engaging the Wisdom of Older Veterans to Enhance VA Healthcare, Research, and Services

**DOI:** 10.1007/s11606-021-07076-x

**Published:** 2022-03-29

**Authors:** Kathryn A. Nearing, Hope M. Adams, James Alsphaugh, Serena E. Douglas, Thomas R. Feller, Robert Fleak, Vernon Moore, Susan Martin-Sanders, Thomas M. Schultz, Karleen Stratton, J. Pat Sullivan, Lance Van Sickle, J. David Yates, Terry A. Yates, Daniel D. Matlock

**Affiliations:** 1 Veterans Affairs Eastern Colorado Geriatric Research Education and Clinical Center, Rocky Mountain Regional VA Medical Center, 1700 North Wheeling Street, Aurora, CO USA; 2 Division of Geriatric Medicine, School of Medicine, University of Colorado Anschutz Medical Campus, Aurora, CO USA; 3 Tarrant County Public Health, Fort Worth, TX USA; 4 VA Eastern Colorado Geriatric Research Education and Clinical Center (GRECC) Older Veteran Engagement Team, Aurora, CO USA; 5 Adult and Child Consortium for Outcomes Research and Delivery Science, University of Colorado Anschutz Medical Campus, Aurora, CO USA

**Keywords:** Veteran engagement, return on investment

## Abstract

**Background:**

Stakeholder engagement helps ensure that research is relevant, clinical innovations are responsive, and healthcare services are patient-centered.

**Objective:**

Establish and sustain a Veteran engagement board involving older Veterans and caregivers to provide input on aging-related research and clinical demonstration projects.

**Design and Participants:**

The Older Veteran Engagement Team (OVET)—a group of eight Veterans and one caregiver who range in age from 62 to 92—was formed in November 2017 and has met monthly since January 2018. The OVET provides feedback on topics that reflect the foci of the VA Eastern Colorado Geriatric Research Education and Clinical Center (GRECC) (e.g., physical functioning, hearing health, and emotional wellness/mental health). Ongoing evaluation documents the return on investment of Veteran engagement.

**Main Measures:**

The OVET member and provider/investigator meeting evaluations with longitudinal follow-up at 6 and 12 months.

**Results:**

Return on investment of Veteran engagement is multi-faceted. For OVET, ROI ranges from grant support to improved healthcare quality/efficiency to social-emotional benefits. To date, funding awards total over $2.3 M for NIH and VA-funded projects to which OVET provided substantive feedback. Documented impacts on healthcare services include reductions in patient wait times, more appropriate utilization of services and increased patient satisfaction. Social-emotional benefits include generativity, as OVET members contribute to improving clinical and community-based supports for other Veterans. The OVET provides an opportunity for older Veterans to share their lived experience with trainees and early career investigators who are preparing for careers serving Veterans.

**Conclusion:**

The OVET is similar to other established stakeholder engagement groups; team members offer their individual viewpoints at any stage of research, clinical demonstration, or quality improvement projects. The OVET provides a mechanism for the voice of older Veterans and caregivers to shape aspects of individual projects. Importantly, these projects support patient-centered care and promote the characteristics of an age-friendly healthcare system.

**Supplementary Information:**

The online version contains supplementary material available at 10.1007/s11606-021-07076-x.

## BACKGROUND AND SIGNIFICANCE

Stakeholder engagement is an essential strategy for improving the quality of research, translation of research evidence into practice, and dissemination of effective models.^1–4^ Veteran engagement within the Veterans Health Administration (VHA) is relatively new.^5–8^ There is growing interest among VA centers/offices, researchers, healthcare providers, and Veterans in models that explore and integrate diverse Veterans’ perspectives to enhance planning, implementation, and dissemination processes.^9,10^

The VA Eastern Colorado Geriatric Research Education and Clinical Center (GRECC) is one of 20 centers of excellence focused on supporting Veterans as they age. Every GRECC has a mission to prepare the clinical and research workforce, develop new or improve existing healthcare services, and conduct aging-related research.^11^ When the Eastern Colorado GRECC was established in October 2014, its leadership shared a commitment to ensuring that older Veterans and caregivers had opportunities to inform the center’s work. The Older Veteran Engagement Team (OVET) was subsequently established in 2017. Coordinated and facilitated by the GRECC Associate Director for Education and Evaluation (KAN), OVET provides a regular mechanism for Veteran and caregiver perspectives to inform all facets of the GRECC mission. Here, we describe methods used to create and sustain this vital aspect of our infrastructure. We highlight the personnel and time commitment required, both of GRECC and OVET members.

Return on investment (ROI) of stakeholder engagement is multi-faceted. We highlight three specific types of ROI for OVET: financial (e.g., research funding), healthcare quality and process improvement (e.g., efficiency, patient experience/satisfaction, and access), and social-emotional (e.g., enhanced social engagement as members give back to other Veterans, and intergenerational connectedness as members support the learning and success of early-career investigators, trainees and fellows). Narrative case examples, drawn from OVET meeting notes and longitudinal surveys, illustrate each type of ROI.

## METHODS

Methods include member recruitment, selection, and orientation; monthly meetings; evaluation and feedback to members; and dissemination of information regarding the impact of Veteran engagement. KAN dedicates .10 FTE to coordination, facilitation, evaluation, and dissemination tasks—the equivalent of approximately $6,000 annually.

### Member Recruitment and Selection

The OVET includes Veterans who receive healthcare in the VA and those who have private insurance. To recruit members, KAN engaged provider networks at the Rocky Mountain Regional VA Medical Center (RMR VAMC) and the Seniors Clinic affiliated with the University of Colorado Hospital. This outreach yielded 25 initial referrals of older Veterans (target age: ≥ 65). KAN or a graduate assistant contacted each referral to provide an overview of the GRECC, the purpose of the engagement team, and to invite each candidate to take part in a 20–30-minute interview. Interviews followed a standardized protocol (Appendix 1), which explored military, civilian, and other relevant life experiences, including prior experience serving on committees or working as part of a team. While participation in research was not a requirement, we explored familiarity or direct experience with research. We asked about experiences navigating differences of opinion as we sought members who could engage in productive conversations where different perspectives were elicited. Finally, we explored issues that might affect participation (e.g., hearing or sight limitations, mobility, access to technology, and preferred ways of receiving information); we used this information to plan meeting logistics and to make accommodations to facilitate participation.

KAN, the graduate student, and a member of the GRECC leadership team with experience establishing a patient engagement board^12^ held a formal candidate selection meeting. Two of these individuals independently reviewed the information available for each candidate. Recommendations and final selections were made through group discussion/consensus, with the goal of maximizing the diversity of the team.

### Member Orientation

**Figure 1:**
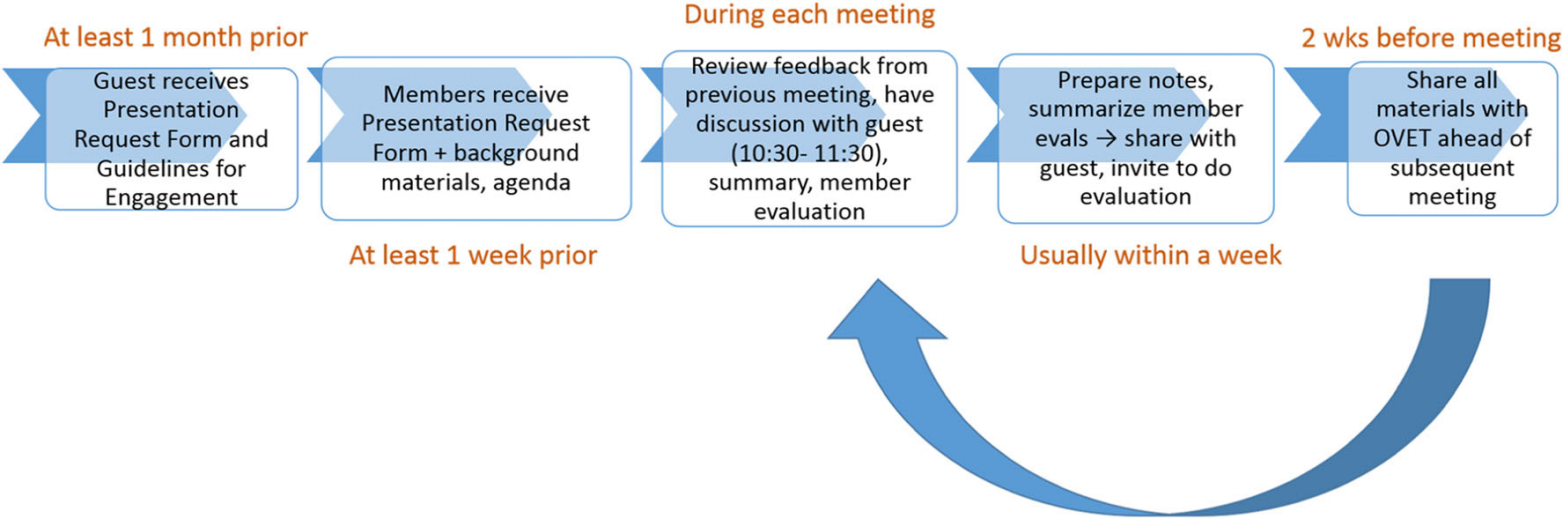
Workflow—what happens before, during, and after each OVET meeting.

During the 4-hour, in-person orientation, we worked to establish group rapport and provide sufficient background information and resources to help prepare members for their roles. We reviewed materials included in OVET orientation packets, which addressed what to expect before, during, and after meetings (Fig. 1), and how members’ feedback/input would be used. In addition, we explored each person’s interest in or motivations for joining the team. Comments most often reflected a desire to help other Veterans. In addition, members wanted to learn about the aging process. Priority topics included the following: dementia and mental health more broadly, access to healthcare services and information, and topics pertaining to the social determinants of health (e.g., transportation and affordable housing for Veterans). Finally, we worked together to establish group norms and drafted a mission statement: “Our mission is to enhance the benefit of research and clinical and community-based services for Veterans through collaboration with Veterans and caregivers.” At the conclusion of the orientation, candidates were invited to review and sign a Member Commitment Letter. This letter specified a commitment of one year and a member stipend ($600). The Veteran Integrated Service Network (VISN) regional office provides funding for stipends – a budgetary request approved through the establishment of a memorandum of understanding each year (total requested in FY21: $6,600).

### OVET Meetings

**Figure 2:**
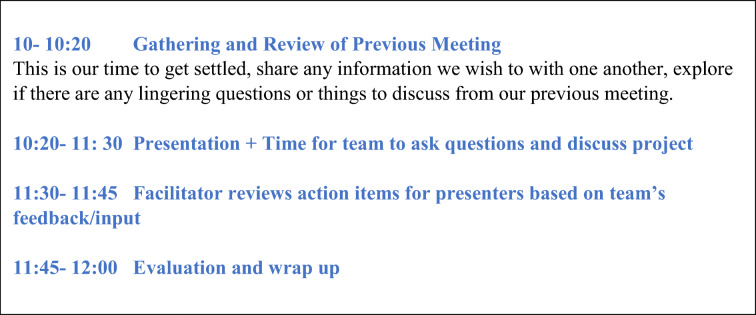
OVET meeting agenda.

Members determined the schedule for monthly 2-hour meetings. Figure 2 presents a typical meeting agenda. After a brief check-in, KAN provides a synopsis of the previous meeting’s topic, highlights of the discussion, and invites reflections/additional ideas that surfaced for members as they reviewed the meeting notes and the previous guest’s evaluation. After the guest for the day’s meeting joins, we do brief introductions. The guest (VA researcher, clinician, or community-based service provider) gives a brief presentation with members invited to ask clarifying questions. Members also have supporting documents, provided prior to the meeting. These materials include a “Presentation Request Form” (Appendix 2), featuring questions that help focus the discussion, and additional background materials. KAN facilitates by keeping the conversation focused on the guest’s questions, summarizing main points, probing for differing viewpoints, and managing time. We devote the last 15–20 min of each meeting to any outstanding business, informal sharing, and completing the brief member evaluation form (Appendix 3).

Prior to the COVID-19 pandemic and the transition to virtual meetings, our team met in-person at the GRECC offices adjacent to the Rocky Mountain Regional VA Medical Center (RMR VAMC). This meeting space was available at no cost with free parking available. KAN provided a simple, nutritious snack (~$15 per meeting). Two members prefer to receive meeting materials via regular mail, with average postage costs of ~$5/month.

### Evaluation

KAN sends detailed notes, a summary of member evaluations, and a brief survey to each guest within 3 weeks of the OVET meeting. The presenter evaluation survey (Appendix 4) invites the guest to share thoughts about how they intend to use the team’s feedback and to identify any feedback they cannot use and the reason/rationale. All information is shared with the team prior to the next meeting for review and discussion. KAN also uses a longitudinal follow-up survey (Appendix 5) to document how the team’s input has informed initiatives affecting older Veterans and caregivers. KAN is present at GRECC meetings during which relevant project updates are often shared. These GRECC meetings provide opportunities to ensure OVET feedback is considered and to listen for changes made to projects in response.

Member meeting evaluations inform immediate, incremental refinements to our processes. In addition, we dedicate one meeting each year—our “Year-in-Review” meeting—to reflecting on the previous year, the topics addressed, and the degree to which these topics reflected the priorities of the VHA, the GRECC, and OVET members. We review information collected from previous guests using the 6- and 12-month follow-up surveys. We identify ways to improve meetings and new topics of interest to members.

### Dissemination

KAN reports OVET activities and concrete ways member input informed research, clinical demonstration projects, and community-based services for older Veterans in annual reports and presentations. Key audiences for these formal reports include executive leaders at the RMR VAMC, the VISN regional office, and the GRECC national office within the VHA Office of Geriatrics and Extended Care. Our work has also been featured in the national GRECC newsletter, *The Forum on Aging*, and through presentations to VA investigators (e.g., RMR VAMC *Research Days* and GRECC-sponsored Grand Rounds).

## RESULTS

### Our Team

The OVET is comprised of eight Veterans (six men and two women) and one caregiver, ages 62 to 92. Veteran members represent three branches of the military and have 0 to 100% service ratings for disability based on diagnosed medical conditions directly attributable to military service. Hearing loss, post-traumatic stress, traumatic brain injury, and mental health challenges are the most common chronic conditions and complex medical issues experienced by members that inform their perspectives. Employment histories span multiple sectors, including government (two were engineers); business (two owned companies); healthcare (includes formal caregiving and directing an emergency department); education; and information technology. Table 1 presents member demographics, which are reflective of the US Veteran population ≥65.

**Table 1:** Current OVET Member Demographics (*n*= 9) Compared to US Veteran Population ≥65

Demographic characteristic		Number (%)	Corresponding % of US Veteran Pop ≥65^1^
Gender	Male	6 (66%)	52.2%
	Female	3 (33%); 2 (22%) are Veterans	18.1%
Role on team	Veteran	8 (89%)	--
	Caregiver	1 (11%)	--
Race*	White	8 (89%)	81%
	Black	1 (11%)	17.8%
	American Indian /Alaska Native	1 (11%)	22.3%
Branch of Military	Army	4 (50%)	44%
	Navy	2 (25%)	21%
	Marine Corps	2 (25%)	11%
Era	Korean War	2 (25%)	7.2% (% of all war-time Veterans)
	Vietnam	4 (50%)	41.2% (% of all war-time Veterans)
Civilian work experience*	Government	3 (66%)	58.9%
	Business owner	2 (22%)	12.8%
	Healthcare provider/administrator	2 (22%)	--
	Information Technology	1 (11%)	--
	Education	1 (11%)	--
	Caregiver (informal)	1 (11%)	--

1 US Veteran Demographic data sources: Population Tables. National Center for Veteran Analysis and Statistics. www.va.gov/vetdata/Veteran_Population.asp. Accessed June 19, 2021. (We used 2020 as the reference year and ≥65 where data by age strata were available.) These data tables were used for white race, period of service and branch of military. US Veteran statistics for gender and work categories were obtained from Profile of Women Veterans: 2015. Prepared by the National Center for Veterans Analysis and Statistics. December 2016. United States Department of Veterans Affairs; US Veteran statistics for race (other than White) were obtained from 2014 Minority Veterans Report. Prepared by the National Center for Veterans Analysis and Statistics. April 2016. United States Department of Veterans Affairs

*Percentages for OVET members do not sum to 100% as more than one option can apply

### Return on Investment

ROI may be assessed using financial metrics (e.g., total funding from grants to which OVET made substantive contributions that enhanced significance, feasibility, quality/rigor), healthcare improvements (e.g., optimization of healthcare services, patient experience, and outcomes), and social benefits (e.g., to OVET members who value opportunities to give back to other Veterans). We use a case example to highlight each type of ROI, organized by topic areas that most often served as the focus of projects reviewed by OVET: physical functioning, hearing health, and mental health. A corresponding table presents an overview of relevant projects and data from meeting notes and longitudinal surveys that document: OVET feedback, changes guests reported they made to projects in response, and outcomes. These tables also highlight the extent to which trainees and fellows have sought the input of OVET to inform their research and clinical demonstration projects. Collectively, these projects support the goals of an age-friendly healthcare system: to promote mentation, mobility, medications (specifically, reductions in polypharmacy), and what matters to the Veteran.^13^

#### Physical Function

The OVET has provided input on multiple initiatives designed to enhance physical functioning in older Veterans. The long-term goals of this body of work are to support independence and avoid (re)hospitalization and institutionalization. Related initiatives and the roles of associated guests are presented in Table 2. Associated research teams often sought OVET input while planning grant applications. Funding awards total over $2.5M to date.

**Table 2:** GRECC Initiatives Promoting Physical Functioning in Older Veterans, OVET Feedback, and Impact 2018–2020

Initiatives (guest)	OVET feedback, response, and current status
Coaching to support walking in Veterans with lower limb amputation (GRECC Advanced Research Fellow) April 2018	**• OVET feedback:** Coaching should encourage social support and environments enjoyable for walking. There are different motivations for initiating versus maintaining walking (or any exercise). Need to tailor coaching accordingly. **• Guest response:** Refined grant, submitted to NIH **• Related outcome:** Application funded in 2019 by the National Institute of Nursing Research (Total 5-year direct cost: $1,250,000; total direct/indirect: $1,942,000)
Walk with Ease program (GRECC Advanced Research Fellow) May 2018	**• OVET feedback:** Major barrier to participating in regular exercise program: inconvenience of travel (drive time and traffic) **• Response:** Added a home-based exercise component. Extended center-based exercise program hours from mid-morning to mid-afternoon (accommodated those who prefer to exercise in morning/afternoon; also avoided rush hour traffic). **• Related outcomes:** Original application not funded; resubmitted June 2021.
Promoting intensive, progressive physical therapy (doctoral student) January 2019	**• OVET feedback:** Noted times when providers could have pushed patients more; shared ideas about ways to make PT more fun/engaging; emphasized importance of helping patients set goals or challenge themselves. Asking about pain can make pain seem worse/direct attention toward it. Instead, ask about mood or tolerance for continuing an activity. **• Response:** Documented in case example in text. **• Related outcome:** OVET feedback informed two successful applications: a VA Office of Rural Health-funded project titled, “Improving Physical Rehabilitation for Rural Veterans with Complex Care Needs“ ($168,153) and a VA SPIRE award ($228,500) from RR&D
Establishing a Fall Prevention Clinic at the RMR VAMC (GRECC Advanced Research Fellow) June 2019	**• OVET feedback:** Make Fall Prevention Clinic interdisciplinary (e.g., include pharmacist and podiatrist); staff with providers knowledgeable about conditions more prevalent in Veterans that can increase fall risk (e.g., TBI). **• Response:** New clinic staffed with a team including a geriatrician, geriatric PT, OT, and geriatric pharmacist. Linked with other programs (e.g., Gerofit) to support fall prevention, enhanced mobility, and independence. Exploring the ability to partner with podiatry. **• Outcome:** New Fall Prevention Clinic opened in May 2021 after delay due to COVID-19.
Aging Veteran Surgical Wellness Program (GRECC Advanced Research Fellow) January 2020	**• OVET feedback:** Engage caregivers as point person for coordinating care with surgical wellness team, including follow-up telehealth appointment after Veteran returns home, and learning/managing associated technological aspects. Validated need to include a pharmacist on surgical wellness team. Encouraged scheduling telehealth check within 2 days of returning home (rather than the 3 days proposed). **• Response:** Refined and implemented program with eight patients before elective surgical procedures were halted due to COVID-19. Continuity of care achieved by interprofessional team who conducts pre-operative assessment to identify vulnerabilities in this population before surgery and provides telehealth follow-up to improve surgical outcomes. Currently analyzing data to assess the impact on ED visits and rehospitalization. **• Outcome:** As a result of this project, RMR VAMC was the first VA to be designated as a Level 1 Geriatric Surgery Center of Excellence.
Tele-rehabilitation Program (doctoral student) June 2020	**• OVET feedback:** Help Veterans set goals and monitor progress. Meaningful goals are tied to activities that contribute to the quality of life (e.g., dancing, gardening, playing with grandchildren). Social connection/social support is really important. Create teams with a team leader who would help motivate and generate friendly competition. **• Response:** Included activity tracking and providing feedback to Veterans regarding progress. Included caregivers to enhance social support and safety. Based on OVET suggestions, will develop training to help caregivers feel more confident and comfortable. Still exploring ways to promote social connections effectively in virtual group exercise sessions; program participant feedback has validated the importance of OVET suggestions in this regard. **• Outcome:** VA Office of Connected Care project, **“**Tele-rehabilitation for Medically Complex Veterans During the COVID-19 Pandemic” funded October 2020 ($169,130)
Emergency Department Weight Management and Exercise Prescription Program – Using ED visits to counsel and connect to services (Emergency Medicine physician, RMR VAMC) October 2020	**• OVET feedback:** Veteran may be in pain, scared, or disoriented and unable to focus on handouts, videos, or even in-person explanations of exercise programs. Offer interactive discussion at ED discharge, after acute needs/concerns have been addressed. Physician can link exercise and weight management to relieving pain. **• Response:** Refined interview guides for patients and providers to include questions re: discharge discussion, including pain management. Created succinct handout with phone numbers for follow-up questions and other VA resources for Veteran to utilize at home. Updated ED discharge instructions to include information regarding transitions of care (i.e., connections to post-ED visit services). **• Outcome:** Study in progress
Effects of exercise on bone health (Advanced Research Fellow planning grant resubmission) November 2020	**• OVET feedback:** Members reviewed research protocol, shared perspectives re: feasibility and burden of participation. Shared that exercise and blood draw requirements would not necessarily be a deterrent (a reviewer concern). However, number of visits and time required may be challenging. Recommended home visits for 24 and 48-hour blood draws and flexibility in scheduling. **• Response:** Reviewers of original proposal expressed concerns regarding tolerance of study participants for exercise regime or required blood draws. OVET members indicated tolerance for both. Based on OVET suggestions, fellow budgeted for home visits for 24 and 48-hr blood draws and to accommodate providing health information (like cholesterol panel) to increase benefits associated with participation. OVET provided signed Letter of Support noting the importance of the proposed research to older Veterans. **• Outcome:** VA Career Development Award resubmitted December 2020.

##### Case Example of Financial Return on Investment

In January 2019, a GRECC clinical investigator and doctoral student (both physical rehabilitation scientists) consulted with OVET in preparation to submit a proposal titled, “Interdisciplinary Mobility Program for Veterans in Skilled Nursing Facilities.” The investigators provided an overview of their research, which demonstrated sustained improvements in physical functioning with rehabilitation approaches that required more exertion and progressive increases in intensity. Their research indicated that the magnitude of improvement in physical functioning was sufficient to maintain independence in the community. The investigators were exploring ways to promote the use of these research findings, particularly to support rehabilitation among Veterans after hospitalization. Their questions for our team included the following: How can rehabilitation therapists engage and motivate patients to participate in more intensive rehabilitation after hospitalization? What are the potential barriers to engaging patients? What are your ideas regarding potential solutions?

During the meeting, we noted times when we thought physical therapists could have pushed patients more. We shared ideas regarding ways staff in post-acute care settings might motivate patients, such as making exercises engaging and fun (e.g., by using music and making activities social). We recommended helping patients set goals to challenge themselves. We noted that one person’s capacity might not be the same as another’s; personalizing goals is also important. The ability to track progress might support engagement and help identify the need to change activities.

Investigators asked for our perspectives regarding ways to address pain or discomfort related to engaging in more intensive physical therapy. They explained that muscle pain (soreness) was to be expected but joint pain indicated a need to modify the activity to avoid injury. One member of our team thought that asking about pain could make the patient more aware of it, which might make their discomfort seem worse just because there was more attention directed toward it. We suggested that, instead of asking about the level of pain, that rehabilitation therapists ask about mood or something that assesses tolerance for continuing a given activity (e.g., “How comfortable does that feel?” “How hard is this activity to do?”). We also noted that it may be important, at the beginning of a therapy session, to ask, “Has anything changed or happened that might impact therapy today?” (e.g., having family visiting that caused a disruption in the patient’s ability to do their exercises regularly or that caused them to feel more tired.) A year later and in response to our feedback, the research team reported:

We worked extensively with our occupational therapy partners to provide education and intervention strategies to tailor high-intensity rehabilitation to an individual’s interests or goals by providing a better link between the therapy exercise/activity and the patient’s interest or goal. We are working with OTs to create an archive of unique and fun ways to engage patients in therapy (e.g., working on pet care, gardening, baking/kneading bread). We are currently working with two therapy teams and encourage the teams to share success stories of unique patient engagement approaches that integrated elements of fun into the therapy session (i.e., music, having a friend complete exercise in tandem). [Your] valuable insight is helping to shape our future research objectives, with patient engagement at the forefront.

#### Hearing Health

The Eastern Colorado GRECC has a robust allied health training program. Audiology is one of five disciplines represented. Audiology training requirements include completing a quality improvement project. Some of these projects have evolved to become clinical demonstration projects that have been formally adopted by the RMR VAMC and disseminated through national professional meetings, the national GRECC newsletter, and peer-reviewed publications. Table 3 presents the hearing health-related projects on which OVET has provided substantive feedback. To highlight the ROI of Veteran engagement to the healthcare system, we provide a case example of an audiology-related project that improved Veteran satisfaction, optimized healthcare utilization, reduced patient wait times, and enhanced clinic capacity.

**Table 3:** GRECC Initiatives Promoting Patient-Centered Care Among Veterans with Hearing Loss, OVET Feedback, and Impact 2018–2020

Initiatives (guest)	OVET feedback, response, and current status
Expanding hearing health resources (Director of RMR VAMC Audiology Department and training supervisor) January 2018	**• OVET feedback:** simplify name of new, proposed walk-in clinic, provide reminders for annual appointments, expand services to long-term care facilities serving Veterans **• Response:** Changed name of clinic and started providing reminders to schedule annual appointments. Was doing hearing aid checks and maintenance in a post-acute and long-term care facility prior to COVID-19. **• Related outcome:** Average of 34 Veterans served in audiology walk-in clinic per day
Skills building support for Veterans newly fitted for hearing aids (Audiology trainee) November 2019	**• OVET feedback:** Took part in a hands-on demonstration of skills-building component using different kinds of hearing aids. Informed the development of a guide for new hearing aid wearers. OVET provided feedback on visuals to include and recommended a “Tips and Tricks” section, offering their own tried-and-true methods. OVET informed questions used during phone follow-up with new hearing aid wearers. **• Response:** Guide and follow-up incorporated all feedback. Suggestion to hold regular group skills-building workshops is on hold due to COVID-19. **• Related outcomes:** See case example featured in text.
Assistive Listening Device Project (Audiology trainee) April 2020	**• OVET feedback:** provided anecdotes highlighting the value of this project, which were featured in proposal used to garner support from medical center director and funding from the regional office. OVET raised concerns about sanitation of devices between use. OVET provided feedback that simplified and clarified questions used to collect Veteran and caregiver feedback. **• Response:** Project was approved in Summer 2020 with VISN funding support ($3560). Disposable headset covers were purchased; devices are included in cleaning protocols implemented between patient visits. **• Related outcomes:** 40 devices are available in various clinic rooms associated with resident clinics, Geriatric Specialty Care clinics and Geriatric ED. Salience within context of COVID-19: masks make hearing and understanding what is said more challenging; assistive listening devices can help overcome this barrier. Anecdote from resident caring for patient who had forgotten hearing aids: “The Veteran loved it, understood how to use it. It took 20 seconds to put it on, adjust the volume to a comfortable level. That 20 seconds saved 10 minutes.”
Incorporating audiologic best practices into residents and Geriatric Medicine Fellows' practice (Audiology trainee) December 2020	**• OVET feedback:** Brainstormed screening questions providers could use to assess hearing loss indirectly (e.g., in case Veteran is reticent to admit hearing loss). Also noted key signs, such as a Veteran turning his/her head a particular way to hear. Recommended signs in clinic rooms: Over 60? Have a hearing screen every two years. **• Response:** Educate providers about objective signs of hearing loss during team huddles before residents and Geriatric Medicine Fellows see patients. Added flyers advertising classes on tinnitus and communication to mailings as OVET members noted that they had benefited from these classes and strongly encouraged broader advertising. **• Related outcomes:** Geriatric Medicine Fellows have reported greater awareness of hearing loss in Veteran patient populations.

##### Case Example: Improved Healthcare Service Quality and Efficiency

In October 2019, an audiology trainee met with OVET seeking input on his quality improvement project. The goal of his project was to improve skills in placing, cleaning, and maintaining hearing aids; a secondary goal was to increase access to information and other sources of ongoing support for Veterans newly fitted with hearing aids to increase utilization of and satisfaction with these assistive devices.

In his review of 104 RMR VAMC audiology department records, he determined that 41% of walk-in appointments stemmed from the lack of knowledge of hearing aid handling and care. Lack of knowledge and basic skills for cleaning and caring for hearing aids contributed to poor performance and inconsistent use of these devices, which are essential to maintaining social connections and cognitive function. In response, this project provided hands-on teaching (pilot tested with OVET), written materials with visual references, and encouraged caregiver involvement in hearing aid fittings. With our feedback, the audiology trainee created a “Hearing Aid Troubleshooting Guide”—a step-by-step guide of what to do at home if a Veteran’s hearing aid is not working. We provided feedback on key visuals to include and recommended adding a “Tips and Tricks” section, which incorporated our tried-and-true methods, for example, of replacing batteries and putting in hearing aids.

Two months after the initial fitting, the audiology trainee planned to follow up with new hearing aid wearers by phone. Our team suggested specific questions to explore during the follow-up call (e.g., What information have you used/needed to refer to? Were the materials helpful? Are you using your hearing aids consistently? Any issues, questions?).

As a result of the improved hands-on training and enhanced support, within 2 months of implementing these changes, the audiology department observed a 44% decrease in the total number of follow-up appointments focused on addressing the lack of basic hearing aid skills. Audiologists also observed a significant increase in the number of Veterans who included family members at fitting appointments. Veterans reported being more satisfied and self-sufficient with their devices. This project has reduced the number of unnecessary follow-up appointments and patient wait times, creating more capacity to care for Veterans with other hearing health needs. Future directions (post-COVID-19) include establishing regular classes for new and established hearing aid wearers as another mechanism to enhance skills and support.

#### Mental Health

From the beginning, members expressed that mental health, in general, and dementia, specifically, should be priority topics for OVET. To respond to the expressed interest both to learn about and help Veterans experiencing related health concerns, KAN has directly outreached to guests whose work focused on these areas. Table 4 presents mental health-related projects. To highlight the social return on investment of Veteran engagement, we provide a case example of the robust feedback provided to inform programs to address loneliness among older adults during the COVID-19 pandemic.

**Table 4:** Initiatives Promoting Mental Health Among Older Veterans, OVET Feedback, and Impact 2018–2020

Initiatives (guest)	OVET feedback, response, and current status
Mental and behavioral health support for older Veterans in rehabilitation (T32 palliative care trainee) March 2019	**• OVET feedback:** Provided feedback on interview guide, especially language and ways to frame/ approach the topics of (previously untreated) mental health with Veterans in non-stigmatizing ways. **• Response:** Incorporated more emphasis on cognition as a component of mental health as a result of the OVET meeting. **• Outcome:** Investigator reported in October 2019, “I completed data collection with providers ... Initial findings helped develop a model of behavioral health in nursing homes ... The OVET group’s insight about attitudes toward behavioral health was spot on and addressed by the providers in my study.”
Connection between hearing health and dementia (Audiology Trainee) December 2019	**• OVET feedback:** OVET suggested a screening process in audiology; should include one or two simple questions for caregivers to assist in timely identification of cognitive impairment **• Response:** Initiated development of brief assessment to use in audiology, with resulting information informing timely referral to gero-psychologist for further evaluation **• Outcome:** Currently on hold due to COVID-19
*Safety in Dementia* caregiver decision aid to address access to firearms (GRECC clinical investigator) October 2019	**• OVET feedback:** Members noted that content related to decisions regarding firearm access/storage options was equally relevant to those with PTSD and TBI. Recommended expanding priority populations to reach with this information (i.e., not solely focus on safety *in dementia*). Some added that Depression, Bipolar and Stroke issues could also affect safety. **• Response:** Based on OVET suggestions, incorporated more language specific to firearm-related issues, namely information on third-party mediators, legal assistance, and information on how to safely transfer firearms to others for safe-keeping during a crisis. **• Outcome:** Clinical demonstration project to test decision aid’s acceptability among older Veterans and caregivers is being planned.
Programming to address social isolation among older adults during COVID-19 (Director, Outreach Programs, Multi-disciplinary Center on Aging) July 2020	**• OVET feedback:** Ideas featured in case example **• Response:** Feedback informed the initial programming of the *Emotional and Mental Health in Older Adults during Challenging Times* Fall and Winter webinar series (weekly one-hour webinars with recordings available) **• Outcome:** Mental Health Wellness series was tremendously successful, with 437 registered to attend these weekly sessions. A Spring 2021 was launched in March 2021.
PTSD and trauma-informed end-of-life care (T32 Palliative Care trainee) August 2020	**• OVET feedback:** Not enough providers ask about mental health. Providers do not use the information in medical record. Discussing mental health is facilitated by having a trusted relationship with provider. Canned (i.e., screening) questions often are asked out of context and by providers who may not have a relationship and who may be in a hurry. Patients want to feel seen, heard, understood. Patients have to self-advocate and be very assertive about having mental health care needs addressed. **• Response:** Use the term "unresolved issues" to invite or frame conversations about trauma, prior life adversity. Include caregivers in my future work in this area. “Really excellent discussion and many good points, but I don’t have the ability to address the system-level issues.” **• Outcome:** As a follow-up to this meeting, invited a VA psychologist and local recovery coordinator to meet with the team. Conversation focused on ways to improve access to mental health services including disseminating information through veteran service organizations.

##### Case Example of Social Return on Investment

In July 2020, the Director of Outreach Programming for the University of Colorado Multi-disciplinary Center on Aging sought input from OVET regarding programs that the center might develop to address loneliness among older adults as part of the center’s multi-pronged response to COVID-19. We expressed particular concern about the increasing rates of suicide among older adults and shared our own experiences during the pandemic. We identified sources of stress, including new and changing ordinances, shifting recommendations, and general confusion. We noted that the changes stemming from Stay-at-Home and Safer-at-Home orders had both positive and negative effects on our daily lives. Benefits, which represented important sources of coping, included extra time to (re)engage in our favorite hobbies, enjoying more time with a spouse/partner, and, for some of us, the ability to remain engaged in regular exercise and volunteer work/service opportunities, including OVET. Negative experiences were associated primarily with the news/social media, with coverage of COVID-19 in combination with politics and social turmoil adding stress to an already difficult situation. We mentioned worries about getting sick and concerns for those without access to technology, which allows individuals to remain socially connected and access resources, such as health/wellness and educational programming. Some members described recognizing signs of depression in neighbors who seemed aimless without more social support and structure. We observed that older adults who were not physically or socially active before COVID-19 may be even less so during the pandemic.

With these perspectives as context, we offered ideas to enhance support for older adults during the pandemic. Ideas included encouraging older adults to set daily/weekly goals, develop hobbies/interests, engage in regular exercise like walking, work on outdoor community projects such as community gardens, start phone trees, tap into free educational programs (e.g., Active Minds—free series of lectures available online), and download e-books available through public libraries and the National Archives. We acknowledged that older adults may have hearing, sight, and cognitive challenges that make it difficult, for example, to concentrate. For these older adults, we emphasized the importance of sharing information about resources such as audio books. We suggested that the center’s “Town Hall” series could include topics to help address feelings of loneliness, such as “mind-body wellness without drugs,” and provide information about opportunities to participate in virtual support groups (e.g., Vietnam Veterans of America’s Buddy Check program), virtual exercise programs, and games (e.g., *Word Games with Friends*, hosting virtual trivia nights, etc.). Recognizing that lack of access to technology limits access to these sources of support for older adults, we emphasized that the center could be a vital resource to help address the digital divide. To underscore the importance of the latter suggestion, one member described visiting a neighbor who had a smart tv but did not know how to use the features to take advantage of the programs available. One member canvased his neighbors to generate still other ideas for the center to consider. An article published 2 months later in *The Gerontologist* highlighted findings that aligned with themes from our meeting. The published survey data highlighted variability in experiences, similar to those expressed by our team and our observations of friends and neighbors. Our recommendations aligned well with the authors’ who noted:

Those most bothered by social isolation may be most helped via a program introducing digital communication or social media platforms …, as long as they are informed by considerations of both health-related (e.g., visual impairment, dexterity limitations) and non-health-related (e.g., digital illiteracy, limited internet access) issues that may influence older adults’ engagement ... Given that distress associated with loneliness and isolation is linked [to] detrimental effects on health and well-being in later life …, finding creative ways to address this issue in the midst of social distancing is essential (45).^14^

## DISCUSSION

Our engagement has improved the relevance of GRECC aging-related research and, therefore, the potential of research to inform clinical and community-based practice. Similarly, our feedback and input on GRECC clinical demonstration projects have contributed to achieving improved patient satisfaction and health outcomes. Mechanisms that support Veteran engagement enhance the capacity of VHA to become an age-friendly health system and *learning* health system.^13–16^

Funders are increasingly calling for “stakeholder engagement” to enhance the feasibility and acceptability of research methods, validity and reliability of research results, and impact of resulting programs/interventions. Established mechanisms for Veteran engagement, supported by standardized procedures, can be an indicator of the research environment or capacity to carry out the proposed research. Veteran engagement can also support education, training, and career development, which are central to the VA’s mission. When a trainee, fellow, or resident meets with OVET, this can be a formative experience with the trainee/fellow learning how pivotal and fundamentally important it is to get out of the lab and talk with patients and caregivers. This experience can shape how they plan and conduct research going forward. Furthermore, having Veterans recognize and validate the value of the research can help sustain an early-career investigator’s motivational energy as they work to secure their first grant. As one GRECC Advanced Research Fellow stated:

I greatly appreciated the participants' identification of barriers, but also their extremely insightful and creative ways of addressing those barriers or concerns. I found the conversation very engaging and fruitful. *Personally, knowing that I have the support of such an engaged group also gave me the confidence to continue to pursue this research.*

### Limitations

Our group is limited in size and scope. We have made an intentional decision to keep the group size under 10 individuals to help ensure that each person has sufficient opportunity to contribute their input, feedback, and ideas. This decision facilitated our work when we transitioned to a virtual meeting platform in response to the COVID-19 pandemic. While our group is reflective of the demographics of older US Veterans, we make no claims that our group nor our input could adequately represent diverse Veteran populations ≥60. While survey data document that our work shaped projects in important ways, the scope of our impact is limited to individual initiatives and individual investigators/research teams. We may evaluate projects, but we cannot enforce or mandate that changes are made in response.

## CONCLUSION

As members of OVET, we are not participating as research subjects. Unlike research volunteers, we contribute our expert opinions to multiple projects and at any stage of research. The engagement relationship is ongoing as we participate in regular meetings with communication occurring in between. The benefits are bi-directional and intergenerational. We find it particularly gratifying to meet trainees and fellows who are preparing for careers to serve Veterans. The OVET provides an opportunity to contribute our lived experience to the work of young scholars and healthcare providers during their training. The OVET has also provided an opportunity to continue to volunteer and remain engaged during the pandemic.

While our team does not claim to represent all Veterans, we have contributed valuable perspectives, potentially beneficial to any Veteran, because of the diversity of our team and the wealth of our ideas. The goal of Veteran engagement, generally, and our team, specifically, is to promote health equity. We want to help address health disparities among Veteran populations so that we do not leave behind our brothers and sisters in arms.

## Supplementary Information

ESM 1 (DOCX 59.6 kb)

## Declarations

### Disclaimer

The contents do not represent the views of the US Department of Veterans Affairs or the United States Government.
