# Supplementary material for: Engaging the Wisdom of Older Veterans to Enhance VA Healthcare, Research, and Services
Source: J Gen Intern Med. 2022 Mar 29;37(Suppl 1):22–32. doi: 10.1007/s11606-021-07076-x (PMC8960672; doi:10.1007/s11606-021-07076-x)
Supplement: Supplementary file 1 — (DOCX 59.6 kb) [file 11606_2021_7076_MOESM1_ESM.docx]

**Appendix 1: Interview Guide Used for Member Selection**

**OVET Selection Interview Guide**

**Last Modified: 12.20.17**

Sample talking points:

- Hi, I’m [state name]. I coordinate the Older Veteran Engagement Team.
- I understand that you learned about our new team from [insert name/role] and are interested in learning more. [pause]
- The Older Engagement Veteran Team (OVET) is a new group that will provide feedback and input to inform and guide the work of a center at the VA.
- This center is focused on supporting veterans as they age and caregivers. It is called the Geriatric Research Education and Clinical Center (GRECC).
- The Older Veteran Engagement Team meets each month to provide feedback and perspectives on education, research and health services. **We want to know if the things that we think will help older veterans and caregivers will actually be helpful.**

[pause for questions and any reactions/comments]

- Does this sound like something you might be interested in participating in?

**[If Yes →]** Great. I have a few questions that I’d like to ask you. These will help me learn more about learn more about you and the experiences/perspectives that you can bring to the team.

[Explore whether he/she has 20-30 minutes now or would prefer to schedule a separate time to go through the questions.]

**[If no →]** I really appreciate your time and interest. I enjoyed talking with you. Can I answer any other questions or provide any additional information?

1. Could you tell me a little about yourself, just in general?

If a Veteran, probe about military and post-military experience.

**If a caregiver**, probe about work and volunteer experience.

| **For Veteran candidates** | **For Caregiver candidates** |
| --- | --- |
| - Can you tell me a bit about your military experience? | - What kind of work did you or do you do? |
| - What kind of work did you do after serving in the military? | - [Only if not obvious from the previous response:] In which sector do you do most of your work, whether volunteer or paid?   Business  Education  Government  Health care  Non-profit  Religious  Service  Other, *please specify*: _________________ |
| - [Only if not obvious from the previous response:] In which sector do you do most of your work, whether volunteer or paid?   Business  Education  Government  Health care  Non-profit  Religious  Service  Other, *please specify*: _________________ |  |

1. What interests you about being on the Older Veteran Engagement Team?
2. Do you have any prior experience serving on a committee like the Older Veteran Engagement Team?

- How about at work or in the community?
- Please share some of your experiences with this/these committee(s)?

1. What has been your experience with the VA?
   - [*If a Veteran*:] What are some of the services you have received?
2. Prior experience with research is not necessary in order to serve on OVET. But, we’re curious if candidates have had any prior research involvement. Have you had any type of research experience?
3. **What special skills, interests or background would you contribute to the group if you were selected to be a part of OVET?**
4. Have you ever been in a group situation in which someone had an opinion that was different than yours?
   - What was the result?
   - What action did you take that helped the group move forward?

**I have a few questions that help ensure that we are including a diverse group of veterans and caregivers. These are voluntary; please know that you can choose not to answer any of these questions.**

**Age:** __________________ **Gender:**  Male  Female

If YES, what branch of the military? (check one)

□Airforce □Army □Coast Guard □Marine Corps □Navy

**Veteran:**  No  Yes

**Caregiver of a Veteran:**  No  Yes

**Race** (check one)**:**

American Indian or Alaska Native  Asian  Black or African American

Native Hawaiian or Other Pacific Islander  White  Other ways you identify yourself? ___________________________

**Ethnicity** (check one)**:**  Hispanic or Latino/a  Not Hispanic or Latino/a

**Service connected:**  No  Yes → *If YES,* what percentage? ______________________  Don’t know

These items will help ensure that we include members from different parts of the state.

**Your zip code: _______________________**

**Do you consider yourself:** □Urban □Suburban or □Rural? (Please check only one.)

**Would any of the following help you participate?** *(Please check all that apply.)*

Transportation to/from meetings  Accommodations for difficulties with hearing

Accommodations for difficulties with seeing (e.g., materials provided in large print, in Braille, etc.)

Other, please explain: ____________________________________________________

1. What else you would like us to know about you?
2. Those are all the questions that I have. Are there any questions that you have for me at this time?

[Express gratitude for their interest and time for the interview. Let him/her know that we will follow up with more information in about a week.]

**Appendix 2: Presentation Request Form**

**Older Veteran Engagement Team (OVET) Presentation Request Form**

**Name:**

**Title/ role:**

**Organization:**

**Email:**

**Descriptive title of project or initiative:**

**Stage or phase of project/initiative** (please check all that apply)

| Generating preliminary ideas | Design and development (working through details of the idea/plan) | Implementation | Evaluation (examining processes, effectiveness) | Dissemination  (sharing results and/or trying to expand efforts to other sites) |
| --- | --- | --- | --- | --- |
|  |  |  |  |  |

**What is the purpose of this project/initiative? What are you trying to do? (Please use language that someone with little or no background knowledge would understand.)**

**Relevance to older Veterans? How will they or their caregivers benefit?**

**What are your main questions for the Older Veteran Engagement Team?**

**What contributions are you seeking from the team?**

**Appendix 3: Meeting Evaluation Form completed by OVET Members**

Presenter: ____________________________ Date: _______________

Topic: ____________________________________________________________

1. **How familiar were you with today’s topic before our meeting?**

| Very familiar | Somewhat familiar | Not at all familiar |
| --- | --- | --- |

1. **How well was the project topic explained by the presenter?**

Very well explained

Somewhat well explained

Not explained well or poorly

Somewhat poorly explained

Very poorly explained

1. **What is something that you now understand very well as a result of the presentation?**
2. **What is something you think could have been explained better? How could that information have been presented more effectively?**
3. **How receptive was the presenter to OVET members’ ideas?**

Very receptive

Somewhat receptive

Neither receptive nor dismissive

Somewhat dismissive

Very dismissive

1. **How interesting did you find today’s project topic?**

Very interesting

Somewhat interesting

Neither interesting nor uninteresting

Somewhat uninteresting

Very uninteresting

1. **What else could we have done to improve this meeting?**

**Appendix 4: Meeting Evaluation Form completed by Guest/Presenter**

**Name:**

**Date of OVET meeting:**

**Title of Project:**

1. **Please list the specific changes you plan to make based on interactions with, and recommendations received from, the Older Veteran Engagement Team:**
2. **If feedback was given that you are not planning to act on, please explain why.**

| Not at all helpful |  |  |  | Very helpful |
| --- | --- | --- | --- | --- |
| 1 | 2 | 3 | 4 | 5 |
|  |  |  |  |  |

1. **On a scale of 1 to 5, with 1 indicating *not at all helpful* and 5 indicating *very helpful*, please rate how helpful you found the interaction with OVET.**

**Please explain your rating:**

1. **On a scale from 0-10, how likely are you to recommend the Older Veteran Engagement Team to a friend or colleague?**

| 0 | 1 | 2 | 3 | 4 | 5 | 6 | 7 | 8 | 9 | 10 |
| --- | --- | --- | --- | --- | --- | --- | --- | --- | --- | --- |
|  |  |  |  |  |  |  |  |  |  |  |

1. **Any suggestions or other comments?**

**Appendix 5: 6-/12-month Evaluation completed by Guest/Presenter**

- - - 1. Please list the specific changes you made or implemented based on the feedback/input you received from the Older Veteran Engagement Team. [meeting notes and initial evaluation feedback are provided]
      2. Are there any (other) updates about your project you would like to share with OVET?
      3. Have you encountered any barriers or challenges in your attempts to incorporate or respond to OVET feedback?

Yes, *please specify*: ________________________________________________

No

- - - 1. Is there anything that you might wish to have the team **review again**?

Yes, in order to solicit additional feedback/input.

Yes, to show how I/my team incorporated or responded to their feedback.

No, not at this time.

- - - 1. At this time, would you find it valuable to have OVET's feedback/input on another project or aspect of your work?

Yes, *please specify* ***topic*** *and the* ***best timing*** *for soliciting their feedback/input (OVET meets monthly):* ________________________________________________

No, not at this time.

- - - 1. Do you have any suggestions of older Veterans who you feel would make a good addition to the Older Veteran Engagement Team (either the Denver-based group or one established to bring together rural-residing Veterans)?

Yes, *please provide contact information if/as appropriate*:

No

- - - 1. Any other comments or feedback for us?

Thank you for your time and for engaging the Older Veteran Engagement Team
